# Effects of Steroids and Tocilizumab on the Immune Response Profile of Patients with COVID-19-Associated ARDS Requiring or Not Veno-Venous Extracorporeal Membrane Oxygenation

**DOI:** 10.3390/membranes11080603

**Published:** 2021-08-09

**Authors:** Vito Fanelli, Giorgia Montrucchio, Gabriele Sales, Umberto Simonetti, Chiara Bonetto, Francesca Rumbolo, Giulio Mengozzi, Rosario Urbino, Costanza Pizzi, Lorenzo Richiardi, Paola Cappello, Luca Brazzi

**Affiliations:** 1 Department of Surgical Sciences, University of Turin, 10124 Torino, Italy; giorgia.montrucchio@unito.it (G.M.); luca.brazzi@unito.it (L.B.); 2 Department of Anaesthesia, Critical Care and Emergency, Città della Salute e della Scienza Hospital, University of Turin, 10124 Torino, Italy; gsales@cittadellasalute.to.it (G.S.); usimonetti@cittadellasalute.to.it (U.S.); cbonetto2@cittadellasalute.to.it (C.B.); rurbino@cittadellasalute.to.it (R.U.); 3 Clinical Biochemistry Laboratory, Città della Salute e della Scienza Hospital, University of Turin, 10124 Torino, Italy; frumbolo@cittadellasalute.to.it (F.R.); gmengozzi@cittadellasalute.to.it (G.M.); 4 Department of Medical Sciences, University of Turin, 10124 Torino, Italy; costanza.pizzi@unito.it (C.P.); lorenzo.richiardi@unito.it (L.R.); 5 Department of Molecular Biotechnology and Health Sciences, University of Turin, 10124 Torino, Italy; paola.cappello@unito.it; 6 Center for Experimental and Medical Research Studies (CeRMS), Città della Salute e della Scienza Hospital, University of Turin, 10124 Torino, Italy

**Keywords:** acute respiratory distress syndrome, COVID-19, steroids, Tocilizumab, lymphocytes, extracorporeal membrane oxygenation

## Abstract

Veno-venous extracorporeal membrane oxygenation (VV-ECMO) is a life-saving rescue therapy in patients with Acute Respiratory Distress Syndrome (ARDS). ECMO has been associated with development of lymphocytopenia that is also common in COVID-19. Hyperinflammation may complicate SARS-CoV-2 pneumonia, prompting therapy with steroids and immunomodulatory drugs. We aimed to evaluate the association of therapies such as steroids and Tocilizumab with trajectories of the total leukocytes, lymphocyte subpopulation count, and inflammatory and fibrinolysis markers in COVID-19-related ARDS, requiring or not VV-ECMO support. The association of the trajectories of the leukocytes, lymphocyte subpopulation count, and inflammatory and fibrinolysis markers with treatment with steroids (**Steroids**), Tocilizumab (**Tocilizumab**), both drugs (**Steroids + Tocilizumab**), and absence of treatment (**No Treatment**) were analyzed using mixed effects regression models, where ECMO was considered as a potential effect modifier. One hundred and thirty-nine leukocyte and eighty-one lymphocyte subpopulation counts were obtained from thirty-one patients who required (VV-**ECMO**, N = 13) or not (**no VV-ECMO**, N = 18) extracorporeal support. In both groups, treatment with Steroids + Tocilizumab was independently associated with a significant reduction of 46% and 67% in total lymphocytes, 22% and 60% in CD3^+^, and 61% and 91% in CD19^+^ (B lymphocytes) compared to those obtained without treatment, respectively. In the no VV-ECMO group, Tocilizumab was associated with a 79% increase in total lymphocytes and with a reduction in procalcitonin compared to no treatment. CD45^+^, CD3^+^CD4^+^ (Th cell), CD3^+^CD8^+^, CD4^+^/CD8^+^, the NK cell subpopulation, neutrophils, monocytes, and basophils were significantly reduced by Steroids + Tocilizumab without an effect modification by VV-ECMO support. In critically ill COVID-19 patients with ARDS, concomitant therapies with steroids and Tocilizumab, beside mitigating the inflammation and fibrinolysis, could reduce the total leukocyte, lymphocyte, and subpopulation count. Moreover, the effect of Tocilizumab in increasing the total lymphocytes and reducing procalcitonin might be blunted by VV-ECMO.

## 1. Introduction

Veno-venous extracorporeal membrane oxygenation (VV-ECMO) is a life-saving rescue therapy in patients with acute respiratory distress syndrome (ARDS) who suffer from refractory hypoxemia [1]. The role of ECMO support for patients with ARDS due to COVID-19 is evolving, becoming more apparent as new evidence is generated and maintaining the traditional inclusion criteria, when appropriate resources are available [2,3,4]. Although crude mortality is yet to be determined with ongoing data collection, recent evidence seems to confirm historical VV-ECMO mortality ranging between 40 and 60% [5,6,7,8,9,10].

Extracorporeal support has been associated with alterations in cell-mediated immunity of adult and pediatric patients that involves neutrophils, monocytes, and lymphocytes [11,12,13,14,15]. These perturbations in the immune surveillance system expose individuals to a higher risk of infection [15] and rapid recovery from lymphocytopenia, after weaning from the by-pass, affects patient prognosis [16].

Effector T cells play a pivotal role in orchestrating the host immune response against the SARS-CoV-2 virus. Severe lymphocytopenia is a common finding in critically ill patients with COVID-19 pneumonia and is associated with disease severity and poor outcome [5,17,18]. SARS-CoV-2-associated hyperinflammation and a cytokine storm define the severity of the lung injury [19], which might be mitigated by steroids and immunomodulatory drugs combination therapy [20]. A high percentage of COVID-19 patients have been treated with different intravenous steroids regimens [6,18,21] and their efficacy appears to be promising, although minimal adverse effects are reported [18,22,23,24,25]. Tocilizumab, an IL-6 receptor blockade, licensed for cytokine release syndrome, is under investigation in patients with COVID-19 pneumonia and elevated IL-6 [26]. Preliminary data have shown that the lymphocyte count went back to normal on the fifth day after treatment with Tocilizumab and abnormally elevated C-reactive protein significantly decreased in most patients [27]. However, a recent randomized placebo-controlled trial showed no benefit on the risk of intubation or death, disease worsening, and time to discontinuation of supplemental oxygen [28].

There are no prospective observational studies exploring the relationship between the host immune response status of SARS-CoV-2-infected patients and immunomodulatory therapies in patients undergoing VV-ECMO support. Although, it is known that decreases in the number and function in some lymphocyte populations raised the issue of close monitoring of patient immunological status during extracorporeal support [29]. We aimed to evaluate the association between immunomodulatory therapies such as steroids and Tocilizumab and trajectories of total leukocyte, lymphocyte subpopulation count, and inflammatory and fibrinolysis markers in patients with COVID-19-associated ARDS requiring VV-ECMO support.

## 2. Materials and Methods

This prospective cohort study was conducted at the ECMO referral center of Città della Salute e della Scienza Hospital in Turin, Italy, from 1 March to 30 April 2020. The hospital institutional review board approved the study using data collected for routine clinical practice and waived the requirement for informed consent (approval number 0028437). We extracted data on all consecutive adult patients with confirmed COVID-19-associated ARDS who required or not VV-ECMO. Patients with a positive virus swab test under protective mechanical ventilation (tidal volume of 6 mL/kg to keep the plateau pressure below 30 cmH_2_O), deep sedation, and muscle paralysis who were not responding to the prone position [30] were considered eligible for VV-ECMO if they had a ratio of partial pressure of arterial oxygen (PaO_2_) to the fraction of inspired oxygen (FiO_2_) less than 80 for more than eight hours or less than 50 for more than three hours. The following criteria were excluded: injurious ventilation at high inspiratory pressures for more than a week, contraindication to systemic anticoagulation with heparin, chronic respiratory failure requiring oxygen therapy or non-invasive ventilation, cancer with a life expectancy of less than 5 years, a moribund patient as judged by the treating physician, and a logistic situation in which the ECMO mobile service was not immediately available [1,7].

The data collected included patients’ demographic information, comorbidities, severity disease, compliance of respiratory system at ICU entry, administration of steroids and of Tocilizumab (an IL-6 receptor blockade), days from onset of symptoms to ICU entry, days from hospital to ICU entry, adoption of rescue therapies (lung recruitment maneuvers, prone position, and inhaled nitric oxide), PaO_2_/FiO_2_ ratio, new diagnoses of bloodstream infection (BSI) and ventilator associated pneumonia (VAP) and aetiologic pathogens [31], and ICU mortality. The leukocyte, lymphocyte, and subpopulation ((CD45^+^, CD3^+^, CD3^+^CD4^+^ (Th cells), CD3^+^CD8^+^, CD4^+^/CD8^+^, CD19^+^ (B lymphocytes), and CD16^+^CD56^+^ (NK cells)) count and inflammatory and fibrinolysis marker (C-reactive protein, C-PR; procalcitonin, ferritin, and D-dimer) concentration were collected at ICU admission and repeated at Day 3, 7, and 14. They were compared in the following conditions: absence of treatment with both steroids and Tocilizumab (**No Treatment**), in the presence of treatment with steroids (**Steroids**), with Tocilizumab (**Tocilizumab**), and with both drugs (**Steroids+Tocilizumab**) in patients who required (**VV**-**ECMO group**) or not (**no VV-ECMO group**) extracorporeal support.

Steroids and Tocilizumab were prescribed at the discretion of the treating physician according to internal protocols. Dexamethasone was intravenously administered at a daily dose of 6 mg for 10 days [21]. Tocilizumab was prescribed at dose of 8 mg/kg (maximal dose 800 mg), repeated after twenty-four hours.

Lymphocyte immunophenotyping was performed by an AQUIOS CL Flow Cytometry System using two separate combinations of four or five murine monoclonal antibody panels, each conjugated to a specific fluorochrome and specific for a different cell surface antigen (Kits Tetra- Panels 1 and 2), as per the manufacturer’s instructions (Beckman Coulter, Inc., Brea, CA, USA) (see electronic Supplementary Material File for more details).

### Statistical Analysis

Continuous variables are presented as medians and interquartile ranges (IQR). Categorical variables are presented as counts and percentages. We compared the medians and percentages between the VV-ECMO and no VV-ECMO groups with rank sum and chi square tests, respectively. We evaluated whether exposure to different treatments (No Treatment, Steroids, Tocilizumab, and Steroids + Tocilizumab) had effects on the trajectories of the total leukocytes, lymphocyte subpopulation count, and inflammatory (C-RP, procalcitonin and ferritin) and fibrinolysis (D-dimer) markers within two weeks using a mixed-effect regression model after log transformation to obtain a normal distribution. Furthermore, we evaluated whether ECMO was a potential effect modifier. SOFA was considered as a potential confounder. In the results section, coefficients were reported as the percentage changes to better show the different treatments’ effect sizes. The coefficients and 95% confidence interval (CI) are reported. Statistical analyses were performed using Stata 13.1/SE (Stata Corporation, College Station, TX, USA).

## 3. Results

A total of thirty-one patients with COVID-19-associated ARDS requiring (N = 13) or not (N = 18) VV-ECMO were included. The baseline characteristics of the patients are shown in Table 1.

Patients treated with ECMO were younger than those treated without ECMO (median age 53 vs. 68; *p* < 0.05). Eighty-four percent of the patients were male. The most common comorbidities were hypertension (45%), obesity (32%), smoking (23%), diabetes (13%), and hypothyroidism (10%). Median days elapsed from onset of symptoms and from hospital to ICU entry were 7 (IQR = 5.5–11) and 2.5 (0.5–6), respectively. Median time spent on invasive mechanical ventilation before VV-ECMO was 7 (3–8) days. Patients received antiviral therapy (39%), steroids (32%), Tocilizumab (52%), and a combination of both (19%). Patients treated with VV-ECMO had more severe organ failure (median SOFA 10 vs. 7; APACHE II 24 vs. 11; and SAPS II 56 vs. 31; all *p* < 0.05) and a lower PaO2/FiO2 ratio at ICU entry than those without ECMO (median PaO_2_/FiO_2_ 64 vs. 132; *p* < 0.05). Eleven (35%), one (3%), and seven (23%) of the thirty-one patients had ventilator associated pneumonia, blood stream infection, and both infections, respectively. There were no significant differences in the proportions of septic shock and infections in both groups, except for the combination of VAP and BSI that was more common in patients with ECMO. ICU mortality was significantly higher in the ECMO compared to the no ECMO group.

**Figure 1:**
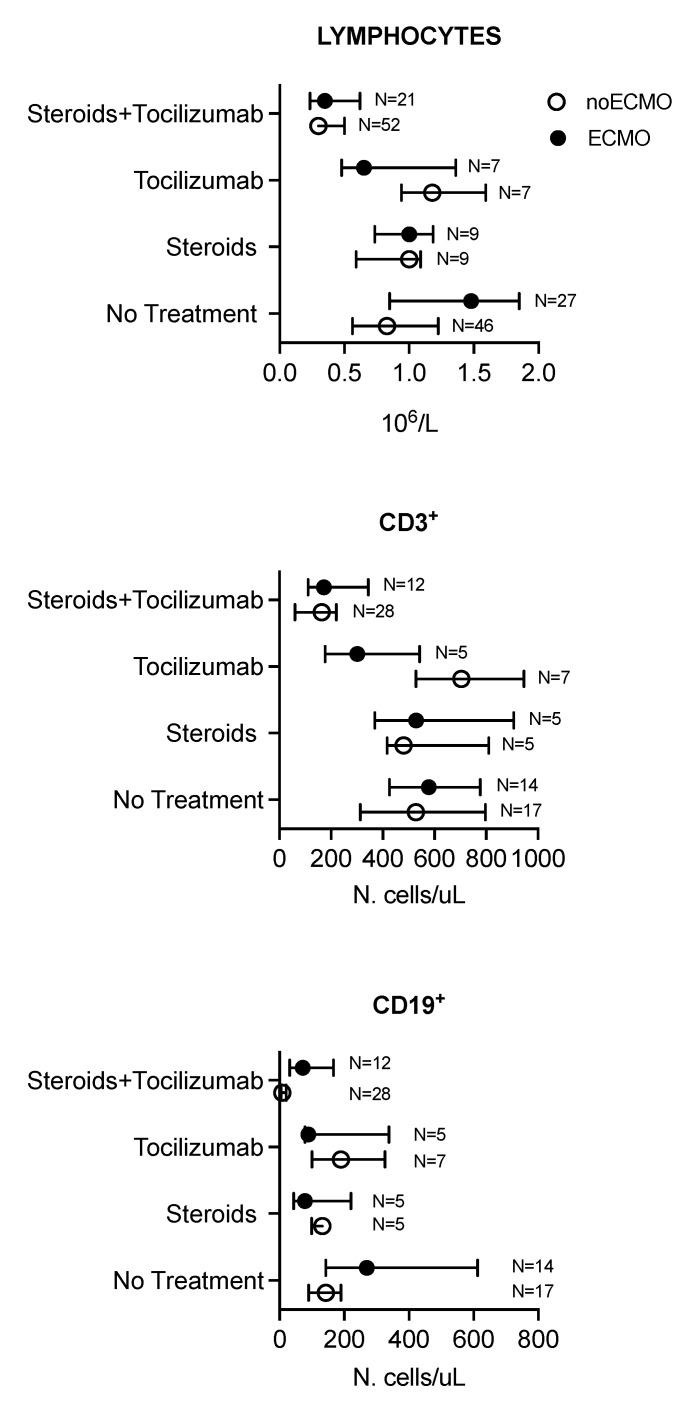
Total lymphocyte, CD3^+^, and CD19^+^ subpopulation counts in the presence or not of steroids and Tocilizumab therapy in COVID-19 patients with ARDS who required or not ECMO support.

**Figure 2:**
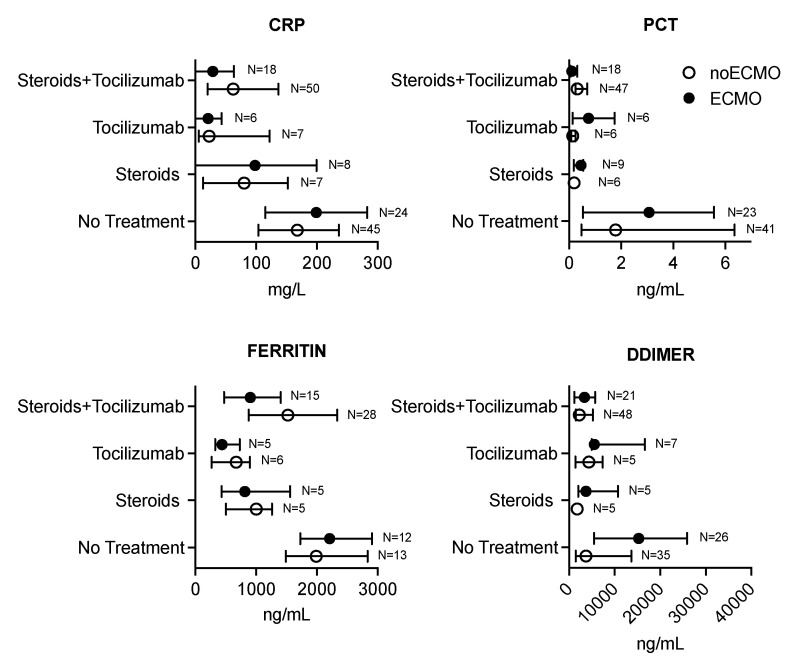
Markers of inflammation with or without steroids and Tocilizumab therapy in COVID-19 patients with ARDS who required or not ECMO support.

One hundred and thirty-nine leukocyte and eighty-one lymphocyte subpopulation counts were performed in two weeks of the follow-up period. The lymphocytes and subpopulation, B lymphocytes, NK cell count, and inflammatory and fibrinolysis markers in both the no VV-ECMO and ECMO groups across the four treatment groups are shown in Figure 1 and Figure 2 and Supplemental Materials Figures S1 and S2.

Evidence of an effect modification by VV-ECMO was observed for the following outcomes: total lymphocyte, CD3^+^, CD19^+^, and procalcitonin, and the stratum-specific effects are shown in Figure 1 and Figure 2. In both the no VV-ECMO and VV-ECMO groups, administration of Steroids + Tocilizumab was independently associated with a significant reduction of 46% and 67% in total lymphocytes (Table 2), 22% and 60% reduction in CD3^+^ (Supplementary Materials File Table S1), and 61% and 91% reduction in CD19^+^ (B lymphocyte) (Supplementary Materials File Table S2), respectively, compared to those obtained without treatment, respectively. 

On the contrary, in the no VV-ECMO group, administration of Tocilizumab was associated with a 79% increase in total lymphocytes compared to no treatment (Table 2). In the no VV-ECMO group, both Tocilizumab and a combination of Steroids + Tocilizumab were significantly associated with a reduction in the inflammatory marker procalcitonin compared to no treatment (Supplementary Materials File Table S3). For the remaining outcomes evaluated, the results from the model without an effect modification by VV-ECMO are shown. The CD45^+^, CD3^+^CD4^+^ (Th cell), CD3^+^CD8^+^, CD4^+^/CD8^+^, NK cell subpopulation, neutrophils, monocytes, and basophils counts were significantly reduced by treatment with Steroids + Tocilizumab without any interaction with VV-ECMO support (Supplementary Materials File Figures S1 and S2 and Supplementary Materials File Tables S4–S12). Combination of Steroids + Tocilizumab was significantly associated with a reduction in the inflammatory marker CRP and D-dimer compared to no treatment (Supplementary Materials File Tables S13 and S14), while ferritin was significantly reduced after exposure to Tocilizumab (Supplementary Materials File Table S15).

## 4. Discussion

This pilot study has demonstrated that in critically ill COVID-19 patients with ARDS, requiring or not VV-ECMO support, concomitant immunomodulatory therapies with steroids and Tocilizumab, in addition to mitigating the inflammation and fibrinolysis, had a significant impact on the reduction in lymphocytes and subpopulation count. Moreover, the effect of Tocilizumab in increasing the total lymphocytes and reducing procalcitonin was blunted during VV-ECMO support.

Virally driven hyperinflammation [32] was the plausible basis to combine antiviral and anti-inflammatory treatments such as steroids and Tocilizumab. Glucocorticoid therapy has been largely prescribed in up to 45% and 70% of hospitalized and critically ill COVID-19 patients, respectively [6,18]. Although, in SARS and MERS patients, corticosteroid therapy delayed viral clearance without effect on mortality [23,33]. Recently, the RECOVERY trial showed that dexamethasone reduced mortality in patients with severe COVID-19 but not in hospitalized patients who did not require supplemental oxygen [21]. In our study, steroids did not differentially affect the leukocyte subpopulation count and inflammatory markers in patients treated or not with ECMO. Tocilizumab, a blocking anti-IL-6 receptor antibody, licensed for cytokine release syndrome, was part of the therapeutic armamentarium in patients with COVID-19 pneumonia and elevated serum IL-6 [26]. In a retrospective study, half of patients treated with Tocilizumab had their lymphocyte count back to normal on the fifth day after treatment. Abnormally elevated C-reactive protein decreased significantly in 84% of patients [27]. However, a recent randomized, double-blind, placebo-controlled trial showed no benefit on the risk of intubation or death, disease worsening, and time to discontinuation of supplemental oxygen [28]. In our study, the steroids and Tocilizumab treatments broadly dampened inflammation and fibrinolysis, as demonstrated by the reduction in plasma levels of C-reactive protein and D-dimer. However, these very effective anti-inflammatory therapies, especially when in combination, were associated with a significant reduction in almost all lymphocyte subpopulations in both patients who required or not ECMO. The robustness of this observation was confirmed by the regression model showing that the combination of the two treatments reduced the lymphocytes. Interestingly, in patients not requiring VV-ECMO, administration of Tocilizumab was independently associated with a 79% increase of total lymphocytes. However, this potential advantage was lost by the combination of steroids and Tocilizumab. This pilot study design cannot fully address the complexity of this question, but we are concerned that profound lymphopenia may expose individuals to the risk of altered viral clearance and of superimposed nosocomial infection that may negatively affect patient outcome. In our small cohort, a significant number of patients treated or not with ECMO had new diagnoses of nosocomial infections (VAP and BSI), complicated by septic shock. Of interest, most of the patients experienced relapse of infections caused by multidrug resistant (MDR) pathogens and two had *Candida* spp. associated BSI, suggesting a profound impairment in immune response [34]. Robust evidence is urgently needed to assess whether systematic corticosteroid and Tocilizumab treatments are beneficial or harmful for SARS-CoV-2 patients. 

ECMO has been progressively offered to severe COVID-19 patients with ARDS who did not benefit from conventional treatment and rescue therapies [6,7,8]. In a mixed adult and pediatric population, extracorporeal support has been associated with several degrees of immune response impairment, consisting of absolute neutrophil, monocyte, and lymphocyte count reductions. The lymphocyte count of the survivors has been reported to reach normal levels within 5 days after weaning from extracorporeal life support, while it remained at low levels in non-survivors [12,13,16]. CD4^+^ T and B cells were reduced during extracorporeal support for cardiac surgery with a return to normal at 24 h after surgery, while the total T lymphocytes did not change [11,12]. For the first time, our study showed that VV-ECMO interacted with anti-inflammatory treatments, decreasing the total lymphocyte, CD3^+^, and B lymphocyte count that orchestrate host immune response against SARS-CoV-2 virus infection. In our cohort, we observed lymphopenia as expected in these very severe patients and more pronounced reduction over time in CD4^+^ T and NK cells, limiting our observation to two weeks. In COVID-19 patients, Th17 cells (CD3^+^CD4^+^) have been described as increased and participating in the cytokine storm. This raises the question of whether ECMO therapy could similarly buffer the increase in Th17 cells in these patients. Of note, the CD8^+^ T cells were not affected over time and deserve to be deeply characterized. A recent report demonstrated the secretion of Granzyme B by CD4^+^ and CD8^+^ T cells isolated from COVID-19 patients although without the co-expression of CD107 and TNFα, suggesting an exhaustion state of these cytotoxic cells [35]. These findings have fostered concern regarding a higher risk of susceptibility to infections, but the implications for selected SARS-CoV-2 patients requiring ECMO are still unknown. 

This study has several limitations. First, the authors acknowledge that this pilot data report a limited number of patients and the effect of such significant impairment of host immune surveillance system is unknown in terms of clinical outcome. However, there are no data of the immune profile response in COVID-19 patients who require ECMO. To note, our population includes VV-ECMO patients only, and we do not know what the effects might have been in patients in veno-arterial ECMO, in whom the immune response may be different. Second, the median follow-up time of two weeks was relatively brief. Third, residual confounding factors might be present as we could only adjust for a limited number of covariates.

## 5. Conclusions

These clinical data from COVID-19 patients with ARDS have shown that a combination of immunomodulatory drugs, such as steroids and Tocilizumab, along with attenuation of the inflammatory and fibrinolysis markers are associated with a reduction in the leukocyte and lymphocyte subpopulation count. In particular, VV-ECMO might be a modifier that blunts the effects of Tocilizumab. Future studies should target these critically ill patients, reporting the clinical outcome as a consequence of therapies affecting innate and adaptative immune responses.

## Figures and Tables

**Table 1:** Baseline characteristics of the Sars-CoV-2 patients treated or not with VV-ECMO.

Variables	All Patients N = 31	ECMO N = 13	No ECMO N = 18
Age, years	59 (53–69)	53 (50–55)	68 (59–73) *
Gender-male, n (%)	26 (84)	10 (77)	16 (89)
BMI	28 (26–31)	28 (28–30)	27 (26–31)
Underlying comorbidities, n (%)			
Obesity	10 (32)	4 (31)	6 (33)
Arterial hypertension	14 (45)	4 (31)	10 (55)
Smoking	7 (23)	1 (8)	6 (33)
Diabetes mellitus	4 (13)	1 (8)	3 (17)
Hypothyroidism	3 (10)	2 (15)	1 (6)
SOFA	8 (6–10)	10 (8–12)	7 (3–10)*
APACHE II	16 (10–23)	24 (21–25)	11 (10–14) *
SAPS II	44 (29–56)	56 (53–59)	31 (29–37) *
Days from onset symptoms to ICU entry	7 (5.5–11)	7 (4–15)	7 (6–10)
Days from hospital to ICU entry	2.5 (0.5–6)	4 (1–13)	2 (0–5)
Days of IMV before ECMO		7(3–8)	
Rescue therapies, n (%)			
Lung recruitment maneuvers	19 (61)	12 (92)	7 (39) *
Prone position	23 (74)	11 (85)	12 (67)
Inhaled nitric oxide	4 (13)	3 (23)	1 (6)
PaO_2_/FiO_2_ at ICU entry, mmHg	83 (62–144)	64 (54–68)	132 (77–185) *
C_RS_ at ICU entry, ml/cmH_2_O	40 (34–50)	36 (24–43)	40 (35–55)
Pharmacologic therapies, n (%)			
Antivirals	12 (39)	9 (69)	3 (17) *
Steroids	10 (32)	2 (11)	8 (61) *
Tocilizumab	16 (52)	6 (46)	10 (56)
Steroids and Tocilizumab	6 (19)	5 (38)	1 (6)
VAP, n (%)	11 (35)	4 (31)	7 (39)
BSI, n (%)	1 (3)	1 (8)	0
VAP and BSI, n (%)	7 (23)	5 (38)	2 (11) *
Septic shock, n (%)	12 (39)	7 (54)	5 (28)
ICU Mortality, n (%)	20 (64)	11 (85)	9 (50) *

List of abbreviations: BMI: body mass index; SOFA: Sequential Organ Failure Assessment; APACHE Acute Physiology and Chronic Health Evaluation; SAPS: Simplified Acute Physiology Score; IMV: Invasive Mechanical Ventilation; ECMO: Extracorporeal Membrane Oxygenation; C_RS_: Respiratory System Compliance. * *p* < 0.05 ECMO vs. no ECMO.

**Table 2:** Mixed-effects linear regression model involving the independent variables related to the log-lymphocyte count in both study groups, adjusted for SOFA.

Variables	Coefficient	(95% Confidence Interval)	*p*
No ECMO group			
Steroids	0.566	−0.234 1.367	0.166
Tocilizumab	0.671	0.274 1.068	0.001
Steroids + Tocilizumab	−0.609	−1.101 −0.117	0.015
ECMO group			
Steroids	−0.284	−0.673 0.104	0.152
Tocilizumab	−0.329	−0.862 0.203	0.225
Steroids + Tocilizumab	−1.106	−1.541 −0.671	0.000

## Supplementary Materials

The following are available online at https://www.mdpi.com/article/10.3390/membranes11080603/s1, Figure S1: CD45^+^, CD3^+^CD4^+^ (Th cell), CD3^+^CD8^+^, CD4^+^/CD8^+^, CD16^+^CD56^+^ (NK cell) subpopulation counts in presence or not of Steroids and Tocilizumab therapy in COVID-19 patients with ARDS who required or not ECMO support. Figure S2: Neutrophils, monocytes, eosinophils and basophils counts in presence or not of Steroids and Tocilizumab therapy in COVID-19 patients with ARDS who required or not ECMO support. Figure S3: Diagram showing patients number and leukocyte count grouping. Table S1: Mixed-effects linear regression model involving independent variables related to log-CD3^+^ cell count in both study groups, adjusted for SOFA. Table S2: Mixed-effects linear regression model involving independent variables related to log-CD19^+^ cell count in both study groups, adjusted for SOFA. Table S3: Mixed-effects linear regression model involving independent variables related to PCT in both study groups adjusted for SOFA. Table S4: Mixed-effects linear regression model involving independent variables related to log-CD45^+^ cell count adjusted for SOFA and ECMO. Table S5: Mixed-effects linear regression model involving independent variables related to log-CD3^+^CD4^+^ cell count adjusted for SOFA and ECMO. Table S6: Mixed-effects linear regression model involving independent variables related to log-CD3^+^CD8^+^ cell count adjusted for SOFA and ECMO. Table S7: Mixed-effects linear regression model involving independent variables related to log-CD16^+^CD56^+^ cell count adjusted for SOFA and ECMO. Table S8: Mixed-effects linear regression model involving independent variables related to CD4^+^/CD8^+^ ratio cell count adjusted for SOFA and ECMO. Table S9: Mixed-effects linear regression model involving independent variables related to log-neutrophils cell count adjusted for SOFA and ECMO. Table S10: Mixed-effects linear regression model involving independent variables related to log-monocytes cell count adjusted for SOFA and ECMO. Table S11: Mixed-effects linear regression model involving independent variables related to log-eosinophils cell count in both study groups adjusted for SOFA and ECMO. Table S12: Mixed-effects linear regression model involving independent variables related to log-basophils cell count, adjusted for SOFA and ECMO. Table S13: Mixed-effects linear regression model involving independent variables related to CRP, adjusted for SOFA and ECMO. Table S14: Mixed-effects linear regression model involving independent variables related to DDimer, adjusted for SOFA and ECMO. Table S15: Mixed-effects linear regression model involving independent variables related to fer-ritin concentration, adjusted for SOFA and ECMO. Table S16: Proportion of antibiotics prescribed before and at ICU admission. Table S17: Proportion of antibiotic molecules prescribed.
