# Supplementary material for: Effects of Steroids and Tocilizumab on the Immune Response Profile of Patients with COVID-19-Associated ARDS Requiring or Not Veno-Venous Extracorporeal Membrane Oxygenation
Source: Membranes (Basel). 2021 Aug 9;11(8):603. doi: 10.3390/membranes11080603 (PMC8399078; doi:10.3390/membranes11080603)
Supplement: Supplementary file 1 [file membranes-11-00603-s001.zip › membranes-1285999-supplementary.pdf]

membranes

MDPI

Supplementary Materials

Effects of Steroids and Tocilizumab on the Immune Response Profile of Patients with COVID-19-Associated ARDS Requiring or Not Veno-Venous Extracorporeal Membrane Oxygenation

Vito Fanelli [1,2,*], Giorgia Montrucchio [1,2], Gabriele Sales [2], Umberto Simonetti [2], Chiara Bonetto [2], Francesca Rumbolo [3], Giulio Mengozzi [3], Rosario Urbino [2], Costanza Pizzi [4], Lorenzo Richiardi [4], Paola Cappello [5,6], Luca Brazzi [1,2]

Citation: Fanelli, V.; Montrucchio, G.; Sales, G.; Simonetti, U.; Bonetto, C.; Rumbolo, F.; Mengozzi, G.; Urbino, R.; Pizzi, C.; Richiardi, L.; et al. Effects of Steroids and Tocilizumab on the Immune Response Profile of Patients with COVID-19-Associated ARDS Requiring or Not Veno-Venous Extracorporeal Membrane Oxygenation. *Membranes* **2021**, *11*, 603. https://doi.org/10.3390/membranes11080603

Academic Editor: Maximilian Valentin Malfertheiner

Received: 19 June 2021
Accepted: 6 August 2021
Published: 9 August 2021

Publisher's Note: MDPI stays neutral with regard to jurisdictional claims in published maps and institutional affiliations.

Copyright: © 2021 by the authors. Submitted for possible open access publication under the terms and conditions of the Creative Commons Attribution (CC BY) license (http://creativecommons.org/licenses/by/4.0/).

1 Department of Surgical Sciences, University of Turin, Italy; vito.fanelli@unito.it, giorgia.montrucchio@unito.it, luca.brazzi@unito.it.
2 Department of Anaesthesia, Critical Care and Emergency - Città della Salute e della Scienza Hospital – University of Turin, Italy; vito.fanelli@unito.it, giorgia.montrucchio@unito.it, gsales@cittadellasalute.to.it, usimonetti@cittadellasalute.to.it, cbonetto2@cittadellasalute.to.it, rurbino@cittadellasalute.to.it, luca.brazzi@unito.it.
3 Clinical Biochemistry Laboratory - Città della Salute e della Scienza Hospital – University of Turin, Italy; gmengozzi@cittadellasalute.to.it, frumbolo@cittadellasalute.to.it.
4 Department of Medical Sciences, University of Turin, Italy; costanza.pizzi@unito.it, lorenzo.richiardi@unito.it.
5 Department of Molecular Biotechnology and Health Sciences, University of Turin, Italy; paola.cappello@unito.it.
6 Center for experimental and Medical Research Studies (CeRMS) - Città della Salute e della Scienza Hospital – University of Turin, Italy; paola.cappello@unito.it.
* Correspondence: vito.fanelli@unito.it.

Methods

Lymphocyte subpopulations were analysed with AQUIOS CL Flow Cytometry system with AQUIOS Tetra Panels (Tetra-1 and Tetra-2) and Software for automated analysis (Beckman Counter, Inc. Ireland). The fluorochrome-conjugated antibodies used for this study are shown in table below.

Marker	Fluorochrome	AQUIOS Panel
CD3	PC5	Tetra-1
CD4	RD1	Tetra-1
CD8	ECD	Tetra-1
CD45	FITC	Tetra-1
CD19	ECD	Tetra-2
CD56+CD16	RD1	Tetra-2

Software algorithms automatically detect the subpopulations of interest. The lymphocyte gate, based on CD45+, electronic volume and light scatter characteristics, is automatically identified and optimized. All cells were then gated for each subpopulation as follow: CD4+ Th cells (CD3+ CD4+); CD8+ T cytotoxic cells (CD3+ CD8+); B cells (CD3– CD19+); NK cells (CD3- CD56/CD16+), as described in figure below and in AQUIOS Tetra System Guide.

CD4+ and CD8+

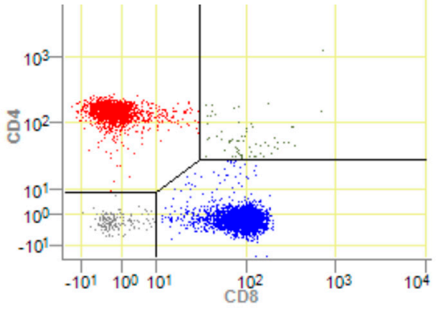

Total lymphocytes

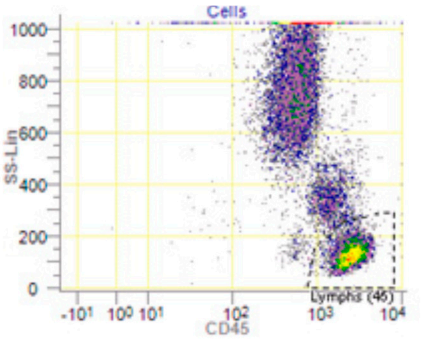

CD3+ subpopulation

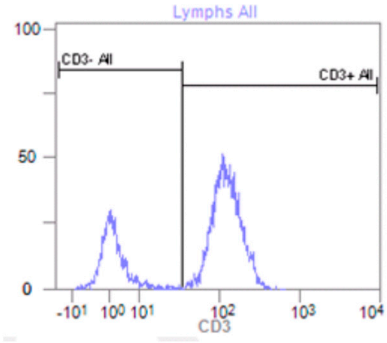

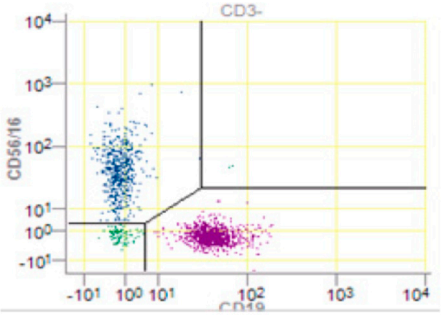

CD456/16+ and CD19+

Table S1. Mixed-effects linear regression model involving independent variables related to log-CD3+ cell count in both study groups, adjusted for SOFA.

Variables	Coefficient	[95% Confidence Interval]		p
**noECMO group**				
Steroids	0.248	-0.603	1.100	0.567
Tocilizumab	0.488	0.041	0.935	0.032
Steroids+Tocilizumab	-0.248	-1.655	-0.621	0.000
**ECMO group**				
Steroids	-0.082	-0.734	0.569	0.804
Tocilizumab	-0.544	-1.263	0.174	0.138
Steroids+Tocilizumab	-0.911	-1.451	-0.371	0.001

Table S2. Mixed-effects linear regression model involving independent variables related to log-CD19$^+$ cell count in both study groups, adjusted for SOFA.

Variables	Coefficient	[95% Confidence Interval]		p
**noECMO group**				
Steroids	0.758	-0.658	2.175	0.294
Tocilizumab	0.905	0.175	1.635	0.015
Steroids+Tocilizumab	-2.459	-3.595	-1.323	0.000
**ECMO group**				
Steroids	-0.471	-1.517	0.574	0.377
Tocilizumab	0.393	-0.775	1.562	0.510
Steroids+Tocilizumab	-0.948	-1.840	-0.056	0.037

Table S3. Mixed-effects linear regression model involving independent variables related to PCT in both study groups adjusted for SOFA.

Variables	Coefficient	[95% Confidence Interval]		p
**noECMO group**				
Steroids	-1.121	-3.785	1.541	0.409
Tocilizumab	-2.105	-3.406	-0.805	0.002
Steroids+Tocilizumab	-3.367	-4.872	-1.862	0.000
**ECMO group**				
Steroids	-0.145	-2.352	2.062	0.897
Tocilizumab	-0.355	-2.842	2.131	0.779
Steroids+Tocilizumab	-0.265	-1.860	1.329	0.744

Table S4. Mixed-effects linear regression model involving independent variables related to log-CD45$^+$ cell count adjusted for SOFA and ECMO.

Variables	Coefficient	[95% Confidence Interval]		p
Steroids	-0.157	-0.713	0.398	0.578
Tocilizumab	0.348	-0.108	0.805	0.135
Steroids+Tocilizumab	-1.252	-1.685	-0.819	0.000

Table S5. Mixed-effects linear regression model involving independent variables related to log-CD3$^+$CD4$^+$ cell count adjusted for SOFA and ECMO.

Variables	Coefficient	[95% Confidence Interval]		p
Steroids	-0.216	-0.819	0.386	0.482
Tocilizumab	0.318	-0.194	0.832	0.223
Steroids+Tocilizumab	-1.176	-1.644	-0.709	0.000

Table S6. Mixed-effects linear regression model involving independent variables related to log-CD3$^+$CD8$^+$ cell count adjusted for SOFA and ECMO.

Variables	Coefficient	[95% Confidence Interval]		p
Steroids	0.130	-0.479	0.741	0.674
Tocilizumab	0.200	-0.276	0.677	0.410
Steroids+Tocilizumab	-0.884	-1.377	-0.390	0.000

Table S7. Mixed-effects linear regression model involving independent variables related to log-CD16$^+$CD56$^+$ cell count adjusted for SOFA and ECMO.

Variables	Coefficient	[95% Confidence Interval]		p
Steroids	-0.204	-0.963	0.555	0.598
Tocilizumab	-0.010	-0.603	0.582	0.973
Steroids+Tocilizumab	-1.462	-2.061	-0.862	0.000

Table S8. Mixed-effects linear regression model involving independent variables related to CD4$^+$/CD8$^+$ ratio cell count adjusted for SOFA and ECMO.

Variables	Coefficient	[95% Confidence Interval]		p
Steroids	-0.662	-2.737	1.412	0.531
Tocilizumab	0.078	-1.820	1.662	0.929
Steroids+Tocilizumab	-0.198	-1.880	1.484	0.817

Table S9. Mixed-effects linear regression model involving independent variables related to log-neutrophils cell count adjusted for SOFA and ECMO.

Variables	Coefficient	[95% Confidence Interval]		p
Steroids	-0.161	-0.551	0.228	0.417
Tocilizumab	-0.320	-0.733	0.092	0.128
Steroids+Tocilizumab	-0.594	-0.845	0.006	0.054

Table S10. Mixed-effects linear regression model involving independent variables related to log-monocytes cell count adjusted for SOFA and ECMO.

Variables	Coefficient	[95% Confidence Interval]		p
Steroids	-0.150	-0.607	0.306	0.519
Tocilizumab	-0.306	-0.669	0.055	0.097
Steroids+Tocilizumab	-0.702	-0.897	-0.248	0.001

Table S11. Mixed-effects linear regression model involving independent variables related to log-eosinophils cell count in both study groups adjusted for SOFA and ECMO.

Variables	Coefficient	[95% Confidence Interval]		p
Steroids	-0.782	-3.430	1.866	0.563
Tocilizumab	1.018	0.003	2.032	0.059
Steroids+Tocilizumab	0.195	-1.594	1.985	0.830

Table S12. Mixed-effects linear regression model involving independent variables related to log-basophils cell count, adjusted for SOFA and ECMO.

Variables	Coefficient	[95% Confidence Interval]		p
Steroids	0.191	-0.443	0.826	0.554
Tocilizumab	0.007	-0.463	0.479	0.974
Steroids+Tocilizumab	-0.653	-1.134	-0.172	0.008

Table S13. Mixed-effects linear regression model involving independent variables related to CRP, adjusted for SOFA and ECMO.

Variables	Coefficient	[95% Confidence Interval]		p
Steroids	-28.897	-89.444	31.648	0.350
Tocilizumab	-119.419	-165.062	-73.775	0.000
Steroids+Tocilizumab	-111.183	-159.254	-63.111	0.000

Table S14. Mixed-effects linear regression model involving independent variables related to DDimer, adjusted for SOFA and ECMO.

Variables	Coefficient	[95% Confidence Interval]		p
Steroids	-10334.26	-24436.24	3767.72	0.151
Tocilizumab	-3625.475	-11080.05	3829.101	0.340
Steroids+Tocilizumab	-11190.21	-19539.15	-2841.26	0.009

Table S15. Mixed-effects linear regression model involving independent variables related to ferritin concentration, adjusted for SOFA and ECMO.

Variables	Coefficient	[95% Confidence Interval]		p
Steroids	-689.636	-1843.685	464.4123	0.242
Tocilizumab	-1117.214	-2084.336	-150.0922	0.024
Steroids+Tocilizumab	-579.401	-1428.472	269.6692	0.181

Table S16. Proportion of antibiotics prescribed before and at ICU admission.

Antimicrobial Therapy	All Patients (N=31)	ECMO (N=13)	noECMO (N=18)
Pre-ICU			
at home, n (%)	22 (71)	10 (77)	12 (67)
at home and general ward, n (%)	27 (87)	13 (100)	14 (78)
ICU admission, n (%)	31 (100)	13 (100)	18 (100)
Empiric therapy, n (%)	29 (94)	12 (92)	17 (94)
Combination therapy (≥2), n (%)	28 (90)	13 (100)	15 (83)

Table S17. Proportion of antibiotic molecules prescribed.

Antibiotic molecules	All Patients (N=31)	ECMO (N=13)	noECMO (N=18)
Beta lactamic	31(100)	13 (100)	18 (100)
Pip/Tazo	10 (32)	7 (54)	3 (17)
Ceftriaxone	9 (29)	0 (0)	9 (50)
Ceftobiprole	6 (19)	2 (15)	4 (22)
Meropenem	4 (13)	3 (23)	1 (5)
Ceft/Avibactam	1 ()	0 (0)	1 (5)
Cefepime	1 (3)	1 (8)	0 (0)
Vancomycin	6 (19)	5 (38)	1 (5)
Linezolid	7 (23)	6 (46)	1 (5)
Daptomycin	1 (3)	0 (0)	1 (5)
Fosfomicyn	1 (3)	0 (0)	1 (5)
Macrolids	2 (6)	0 (0)	2 (10)
Levofloxacin	1 (3)	1 (8)	0 (0)
Antifungal therapy	8 (26)	4 (31)	4 (22)

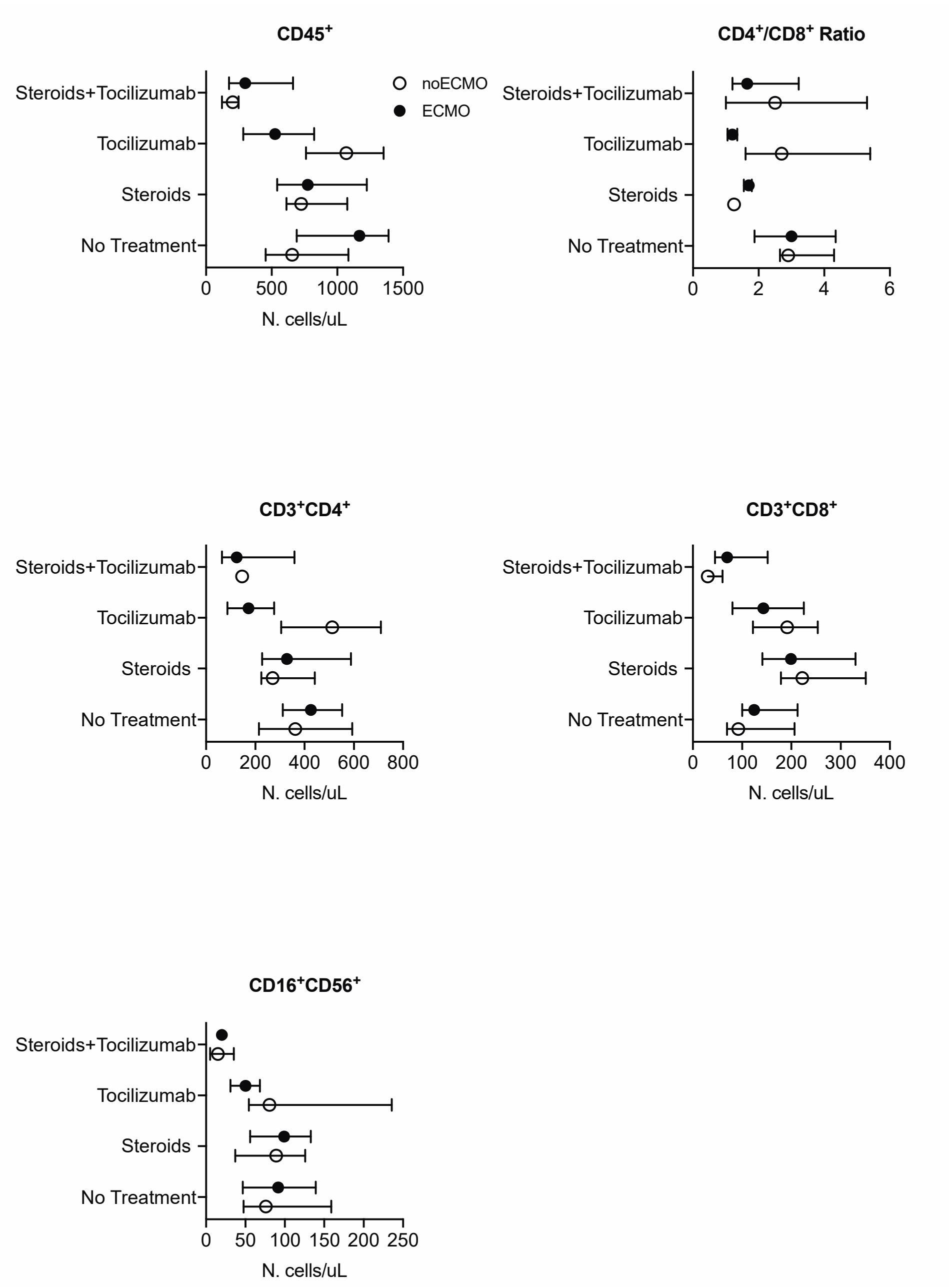

Figure S1. CD45+, CD3+CD4+ (Th cell), CD3+CD8+, CD4+/CD8+, CD16+CD56+ (NK cell) subpopulation counts in presence or not of Steroids and Tocilizumab therapy in COVID-19 patients with ARDS who required or not ECMO support.

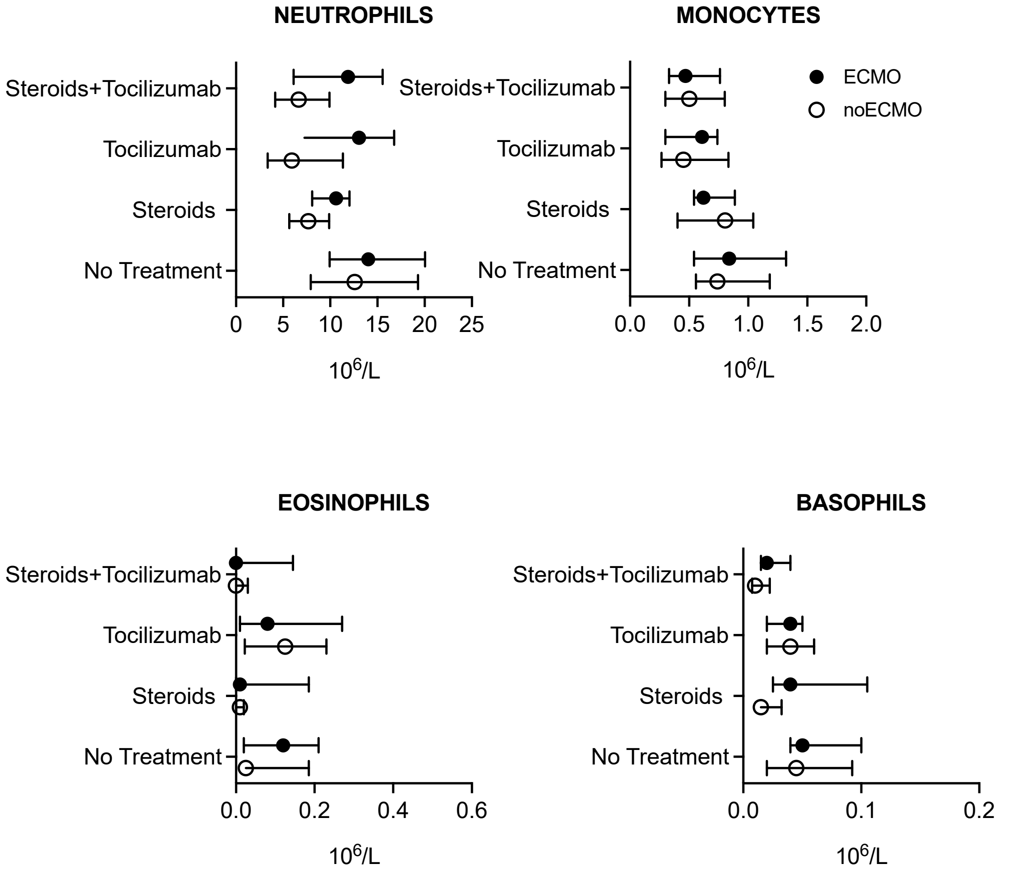

Figure S2. Neutrophils, monocytes, eosinophils and basophils counts in presence or not of Steroids and Tocilizumab therapy in COVID-19 patients with ARDS who required or not ECMO support.

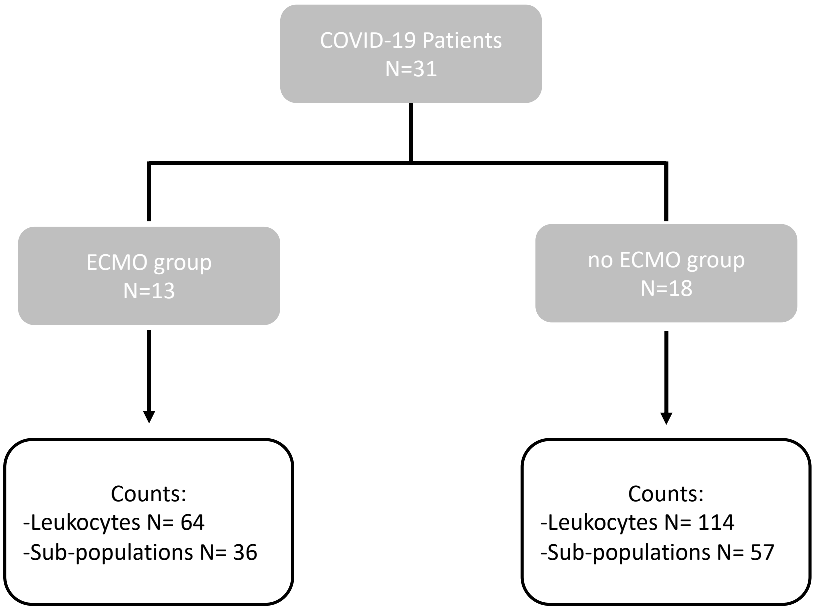

Figure S3. Diagram showing patients number and leukocyte count grouping.
